# Evaluation of Diagnostic Tools of Sarcopenia in Chronic Liver Disease: A Cross-Sectional Observational Study

**DOI:** 10.7759/cureus.100548

**Published:** 2026-01-01

**Authors:** Venkata Koti Reddy Chennapareddy, Shanmughanathan Subramanyam, AK Koushik, Arikila Mounika, Dasari Sai Sarath, Farhanulla Basha, Nirupam Nadella

**Affiliations:** 1 Department of Medical Gastroenterology, Sri Ramachandra Institute of Higher Education and Research, Chennai, IND; 2 Department of Diagnostic Radiology, Sri Ramachandra Institute of Higher Education and Research, Chennai, IND; 3 Department of General Medicine, Dr. D. Y. Patil Medical College, Hospital and Research Centre, Dr. D. Y. Patil Vidyapeeth (Deemed to be University), Pune, IND

**Keywords:** cirrhosis, hgs, lfi, pmi, sarcopenia

## Abstract

Introduction

Sarcopenia is consistently recognized as a strong predictor of poor outcomes in chronic liver disease and is associated with increased mortality in patients with liver cirrhosis. There is limited data on the evaluation and diagnostic tools of sarcopenia in patients with liver cirrhosis in our study setting.

Aim

This study aims to evaluate different diagnostic tools of sarcopenia in patients with liver cirrhosis in our tertiary care center.

Materials and methods

This is a cross-sectional observational study comprising 50 patients with liver cirrhosis, with or without complications. A plain CT scan of the abdomen at the third lumbar vertebra was used to measure the cross-sectional area of the psoas muscle, which was then expressed as the psoas muscle index (PMI) in mm^2^/m^2^. The PMI values of 295 mm²/m² in women and 356 mm²/m² in men were used as cut-off values to define sarcopenia. Handgrip strength (HGS) and liver frailty index (LFI) were measured, and the cut-off values for both diagnostic modalities were determined using receiver operating characteristic (ROC) analysis.

Results

The mean age of the patients was 58.16 years, with male predominance (76%). The prevalence of sarcopenia in patients with liver cirrhosis was 24 (48%). The mean PMI value was significantly lower (p = 0.001) in patients with sarcopenia than in patients with non-sarcopenia (287.28 versus 502.4), suggesting a strong association between PMI and sarcopenia. The ROC curve analysis for HGS as a predictor of sarcopenia revealed an area under the receiver operating characteristic (AUROC) of 1.000 and 0.903, indicating strong diagnostic accuracy in female (p = 0.004) and male (p = 0.001) patients. The optimal cut-off values of HGS for predicting sarcopenia were determined to be 17.3 kg and 26.1 kg, with sensitivities (Sn) of 100% and 94.7% and specificities (Sp) of 85.7% and 63.7% for female and male patients, respectively. Similarly, the ROC curve analysis for LFI as a predictor of sarcopenia revealed an AUROC of 0.985, a cut-off value of 4.2, and a sensitivity of 91.7% and a specificity of 92.3%.

Conclusion

The prevalence of sarcopenia was 48% in patients with liver cirrhosis in our study setting. The optimal cut-off values of HGS and LFI for predicting sarcopenia were found to be 17.3 kg (women) and 26.1 kg (men) and 4.2, respectively, with a good sensitivity and specificity. Indian-specific cut-offs should be used to define sarcopenia, as Western cut-offs may lead to overestimation.

## Introduction

Age-related illnesses are on the rise as the world's population ages. Among these is sarcopenia (from the Greek words "sarx" and "penia"), which is a geriatric disease characterized by the progressive loss of muscular mass and function. It is a crucial indicator of survival and the possibility of complications, particularly for specific patient populations, including those with metabolic diseases and liver cirrhosis [1,2].

Sarcopenia is defined as a generalized loss of muscle mass and strength, which may occur as a primary process related to aging or as a secondary outcome of acute or chronic illnesses, including chronic liver disease [3]. The pooled prevalence of sarcopenia among patients with cirrhosis is estimated at 37.5%, and it has been identified as an independent predictor of mortality [2]. It is consistently regarded as a negative prognostic factor in chronic liver disease, being associated with increased mortality among patients with cirrhosis and hepatocellular carcinoma and those awaiting liver transplantation [4]. Cirrhosis itself is a significant predisposing factor for malnutrition and sarcopenia [5]. Furthermore, the coexistence of sarcopenia in individuals with cirrhosis increases the risk of infection, falls, decompensation, and death [6-8].

Several pathophysiological mechanisms underlie sarcopenia in cirrhosis, including malnutrition, disrupted skeletal muscle protein metabolism, iatrogenic factors, and systemic inflammation [9,10]. The diagnosis of sarcopenia rests on three principal components: diagnostic criteria, assessment modality, and established cut-off values [11].

Handgrip strength (HGS) is a cost-effective, reliable, noninvasive, and reproducible bedside measure for evaluating nutritional status in patients with liver cirrhosis. It serves as a predictor of mortality for those on the transplant waitlist [12]. According to the European Working Group on Sarcopenia in Older People 2 (EWGSOP2) criteria, an HGS cut-off of <27 kg for men and <16 kg for women is used for sarcopenia patients. HGS values differ by ethnicity and age [13,14], with Southeast Asians exhibiting lower HGS than healthy populations in Europe and North America [15]. Therefore, sarcopenia-specific HGS cut-offs in Indian patients with cirrhosis should be derived from normative values obtained from a representative healthy population [14,16].

The psoas muscle index (PMI), defined as the psoas muscle area (PMA) at the L3 vertebral level, is employed for sarcopenia diagnosis and, when adjusted for stature, is referred to as L3 PMI [17]. Additionally, the psoas muscle cross-sectional area has been used to assess sarcopenia [18]. These measurements are quick and simple, do not require specialized software, and have demonstrated reliability as predictors of mortality in liver cirrhosis [19].

Sarcopenia is a key component of frailty [20]. The considerable overlap between sarcopenia and frailty in chronic liver disease gives rise to a condition termed physical frailty [21]. The liver frailty index (LFI) incorporates individual measures such as handgrip strength, chair stands, and balance tests drawn from established instruments. Its main advantages include the ease of integration into clinical practice, excellent interrater reliability and reproducibility, and suitability for longitudinal assessment [22]. As the LFI becomes more widely adopted [23,24], identifying clinically meaningful cut-offs for use in decision-making by clinicians is increasingly important [22].

Patients with advanced liver disease are known to have higher rates of morbidity and death when they suffer from severe malnutrition, as evidenced by sarcopenia and malnutrition [25]. It is widely acknowledged that nutrition plays a significant role in patients with liver disease and transplant candidates, and top authorities have issued updated recommendations [3,26]. However, these recommendations cannot be directly applied to Indians, which presents unique considerations for assessing nutritional status and managing malnutrition [16].

Furthermore, there is limited data regarding the assessment and diagnostic tools for sarcopenia in patients with liver cirrhosis in our setting. Therefore, the present study was conducted for the purpose of evaluating various diagnostic tools for sarcopenia in patients with liver cirrhosis at our tertiary care center.

The primary objective was to assess the sarcopenia predictive ability of LFI and HGS indices in patients with liver cirrhosis. The secondary objective was to determine the association of PMI with sarcopenia in patients with liver cirrhosis and to compare calorie consumption between patients with sarcopenia and non-sarcopenia with liver cirrhosis.

## Materials and methods

This is a cross-sectional observational study conducted at the Department of Gastroenterology, Sri Ramachandra Institute of Higher Education and Research, Chennai, Tamil Nadu, India. The study was performed over a period of 20 months from September 2023 to May 2025 after obtaining ethics committee approval from the Institutional Research Ethics Committee of Sri Ramachandra Institute of Higher Education and Research (approval number: CSP-MED/25/JAN/116/118).

Sample size calculation

A convenient sampling method was employed for this study. A total of 50 patients with liver cirrhosis were recruited. The formula used to calculate the sample size for a population proportion was as follows: N = (Z_(α/2)² * p * (1 - p)) / d². By substituting the values in Table 1 into the formula, the calculated sample size is 48, rounded off to 50.

**Table 1:** Data used to substitute in the normal approximation sample size formula for proportions

Data used as substitute		Values
p	Prevalence of sarcopenia in subjects with liver cirrhosis [17]	0.7
α	Confidence level	0.95
Z	Z value associated with confidence	1.96
d	Absolute precision assumed	0.13
N	Minimum sample size	48

Inclusion criteria

Patients with liver cirrhosis aged 18 years or older, of both genders, and with or without complications of chronic liver disease were included.

Exclusion criteria

Pregnant women and patients with malignancies (hepatocellular carcinoma and cholangiocarcinoma), comorbidities (chronic renal failure and heart failure), and joint disabilities (rheumatoid arthritis and systemic lupus erythematosus) were excluded.

Data collection

A comprehensive demographic history was obtained for each participant. Routine diagnostic and treatment-related investigations were performed, including complete blood count, viral markers (hepatitis B surface antigen {HbsAg} and hepatitis C virus {HCV}), liver function tests, coagulation profile, and other standard laboratory assessments. Body mass index (BMI) and Child-Turcotte-Pugh (CTP) classification were determined for all patients.

**Figure 1:**
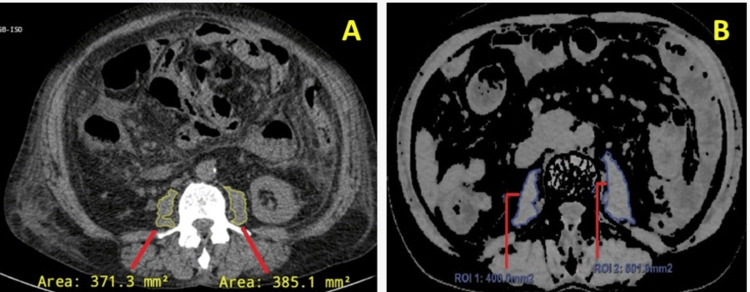
The representative CT images of two cases showing PMA in patients with liver cirrhosis (A) Sarcopenia and (B) non-sarcopenia PMA: psoas muscle area

Following this, CT of the abdomen from the diaphragm to the symphysis pubis was obtained with detector collimation of 24 mm, gantry rotation of 0.5 seconds, pitch factor of 0.938:1, and slice thickness of 2 mm. A single breath hold of 5-9 seconds was used for scanning. Data was transported to a Philips advanced workstation for multiplanar reconstruction (MPR) picture post-processing. Advantage Windows Area Analysis software (GE HealthCare, Chicago, IL) analyzed the psoas muscle regions from CT images and the total psoas muscle area at the L3 vertebral level in the first segment, where both of the vertebrae's transverse processes are visible. The threshold has been set between -30 and +110 HU; this permits the exclusion of fatty infiltration sites and vasculature. Using a region of interest (ROI) free form, the psoas muscle was segmented by tracing its lines on the chosen slice, and the area was measured by clicking the right psoas muscle in the window. Similarly, the left psoas muscle area was calculated. The areas of both left and right psoas muscles were then summed and divided by the patient's height (m²). The PMI values of 295 mm²/m² in women and 356 mm²/m² in men were used as cut-off values to define sarcopenia [17]. The representative CT images of two cases showing PMA in patients with liver cirrhosis are illustrated in Figure 1.

The liver frailty index (LFI) comprises the following three performance-based assessments: (1) handgrip strength: measured in the dominant hand using a hand dynamometer, averaged over three trials (values); (2) timed chair stands: recorded as the number of seconds taken to complete five chair stands with arms crossed across the chest; and (3) balance testing: measured as the duration (in seconds) a patient can maintain balance in three positions: feet side by side, semi-tandem, and tandem for up to 10 seconds each.

These tests were conducted by trained study personnel, and the three components were integrated into the LFI using the calculator available at http://liverfrailtyindex.ucsf.edu [22].

Handgrip strength (HGS) was assessed using a digital hand dynamometer [27]. Measurements were taken with the patient standing, forearm away from the body at thigh level. The participants performed three maximal grip attempts with each hand, allowing at least a 30-second rest between trials. The HGS value was defined as the highest measurement obtained from the dominant hand [28].

Statistical analysis

The data was entered in Microsoft Excel 2021 (Microsoft Corp., Redmond, WA), and statistical analysis was carried out using Statistical Package for the Social Sciences (SPSS) version 23 (IBM Corp., Armonk, NY). Categorical data was represented as frequencies and percentages, and quantitative data was represented as means with standard deviations. Inferential statistics, such as the chi-square test, were applied to qualitative variables to assess association. An independent samples t-test was used to compare quantitative parameters between the groups. The receiver operating characteristic (ROC) curve was computed to determine the cut-off values and the sensitivity (Sn) and specificity (Sp) of HGS and LFI in predicting sarcopenia. P ≤ 0.05 was considered statistically significant.

## Results

Table 2 summarizes the demographic characteristics of the patients. The mean age of the patients was 58.16 years, with male predominance (38, 76%), compared to women (12, 24%). The majority of the patients were in the >60 years of age (27, 54%), followed by 41-50 years (11, 22%), 51-60 years (eight, 16%), and 31-40 years (four, 8%).

**Table 2:** Demographic characteristics SD: standard deviation

Characteristic	Values
Age (years)	
31-40	4 (8.0)
41-50	11 (22.0)
51-60	8 (16.0)
>60	27 (54.0)
Mean ± SD	58.16 ± 11.02
Gender	
Male	12 (24.0)
Female	38 (76.0)

Table 3 compares the BMI, HGS, and LFI values between patients with and without sarcopenia. Patients with non-sarcopenia had a significantly higher mean BMI compared to those with sarcopenia (24.93 versus 18.30; p = 0.001). Similarly, HGS was significantly higher in patients with non-sarcopenia than in patients with sarcopenia (26.33 versus 18.20; p = 0.001). The LFI value was significantly higher in patients with sarcopenia than in patients with non-sarcopenia (4.70 versus 3.30; p = 0.001).

**Table 3:** Comparison of BMI, HGS, and LFI based on sarcopenia Values were expressed as mean ± SD. Sarcopenia: n = 24; non-sarcopenia: n = 26. P-value based on an independent samples t-test. Units of measurement: BMI, kg/m²; HGS, kg, and LFI, unitless (numerical score). Significant: p < 0.05 BMI, body mass index; HGS, handgrip strength; LFI, liver frailty index; SD, standard deviation

Parameters	Sarcopenia	Mean	Mean difference	P-value
BMI	Non-sarcopenia	24.93 ± 4.54	6.62	0.001
	Sarcopenia	18.30 ± 2.14		
HGS	Non-sarcopenia	26.33 ± 4.88	8.12	0.001
	Sarcopenia	18.20 ± 5.00		
LFI	Non-sarcopenia	3.30 ± 0.49	-1.40	0.001
	Sarcopenia	4.70 ± 0.35		

Table 4 presents the association of viral markers (HbsAg and HCV) and CTP classification with the presence of sarcopenia. The analysis revealed no statistically significant association between these variables and sarcopenia (p > 0.05 for all).

**Table 4:** Association of viral markers and CTP classification with sarcopenia Significant: p < 0.05 HbsAg, hepatitis B surface antigen; HCV, hepatitis C virus; CTP, Child-Turcotte-Pugh

			Non-sarcopenia	Sarcopenia	Total	Chi-square value	P-value
HbsAg	Non-reactive	Count	20	18	38	0.02	0.874
		%	76.9	75.0	76.0		
	Reactive	Count	6	6	12		
		%	23.1	25.0	24.0		
HCV	Non-reactive	Count	26	22	48	2.25	0.133
		%	100.0	91.7	96.0		
	Reactive	Count	0	2	2		
		%	0.0	8.3	4.0		
CTP	A	Count	4	1	5	4.42	0.109
		%	15.4	4.2	10.0		
	B	Count	12	7	19		
		%	46.2	29.2	38.0		
	C	Count	10	16	26		
		%	38.5	66.7	52.0		

Table 5 compares the mean PMI values between patients with sarcopenia and non-sarcopenia. Patients with sarcopenia had a significantly lower mean PMI value compared to those without sarcopenia (287.28 versus 502.4; p = 0.001).

**Table 5:** Comparison of PMI Values were expressed as mean ± SD. Sarcopenia: n = 24; non-sarcopenia: n = 26. P-value based on an independent samples t-test. Significant: p < 0.05 PMI, psoas muscle index; SD, standard deviation

Sarcopenia	Mean	Mean difference	P-value
Non-sarcopenia	502.40 ± 112.45	215.12	0.001
Sarcopenia	287.28 ± 45.99		

**Figure 2:**
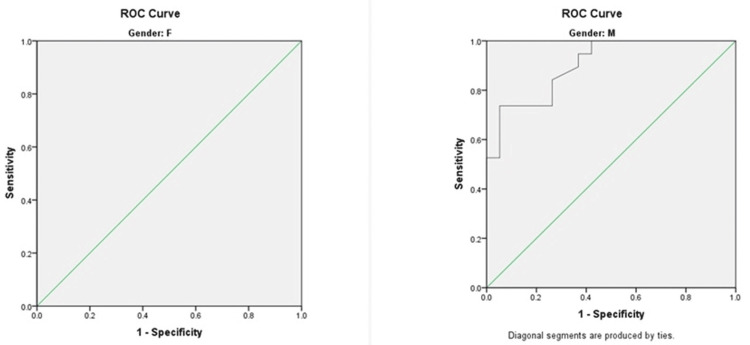
The ROC curve analysis for predicting sarcopenia using HGS as a predictor in female (F) and male (M) patients ROC, receiver operating characteristic; HGS, handgrip strength

The ROC curve analysis for HGS as a predictor of sarcopenia revealed an area under the receiver operating characteristic (AUROC) curve of 1.000 and 0.903, indicating strong diagnostic accuracy in female (p = 0.004) and male (p = 0.001) patients. The optimal cut-off values of HGS for predicting sarcopenia were determined to be 17.3 kg and 26.1 kg, with Sn of 100% and 94.7% and Sp of 85.7% and 63.7% for female and male patients, respectively, as shown in Figure 2 and Table 6.

**Table 6:** The AUROC value for predicting sarcopenia using HGS as a predictor *Significant: p < 0.05 CI, confidence interval; HGS, handgrip strength; AUROC, area under the receiver operating characteristic

Parameter	Gender	Area	Standard error	P-value	Asymptotic, 95% CI	
					Lower bound	Upper bound
HGS	Female	1.000	0.000	0.004*	1.000	1.000
	Male	0.903	0.047	0.001*	0.811	0.995

**Figure 3:**
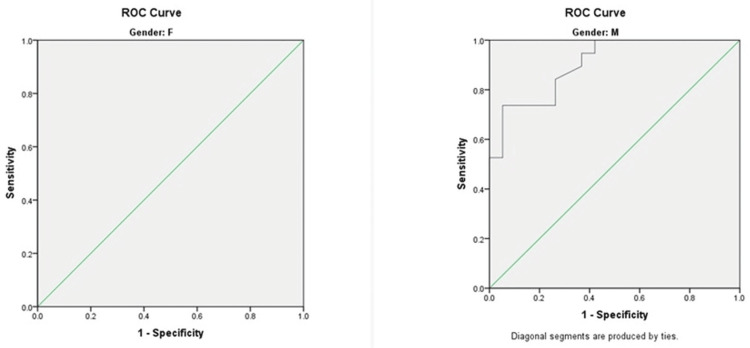
The ROC curve analysis for predicting sarcopenia using LFI as a predictor ROC, receiver operating characteristic; LFI, liver frailty index; F, female; M, male

The ROC curve analysis for LFI as a predictor of sarcopenia revealed an AUROC value of 0.985, indicating strong diagnostic accuracy (p = 0.001). The optimal cut-off value for predicting sarcopenia was determined to be 4.2, with Sn and Sp of 91.7% and 92.3%, respectively (Figure 3 and Table 7).

**Table 7:** The AUROC value for predicting sarcopenia using LFI as a predictor Significant: p < 0.05 AUROC, area under the receiver operating characteristic; LFI, liver frailty index; CI, confidence interval

Parameter	Area	Standard error	P-value	Asymptotic, 95% CI	
				Lower bound	Upper bound
LFI	0.985	0.013	0.001	0.960	1.000

Table 8 compares the mean kilocalorie (kcal) consumption and calorie deficit between patients with sarcopenia and non-sarcopenia. The patients with sarcopenia consumed significantly fewer calories than patients with non-sarcopenia (1035.63 versus 1559.62; p < 0.001). The mean calorie deficit was 543.54 kcal in the patients with sarcopenia and 438.46 kcal in the patients with non-sarcopenia, with a mean difference of -105.08 kcal (p = 0.005).

**Table 8:** Comparison of kilocalorie consumption and calorie deficit based on sarcopenia Values were expressed as mean ± SD. Sarcopenia: n = 24; non-sarcopenia: n = 26. P-value based on an independent samples t-test. Significant: p < 0.05 SD: standard deviation

Parameters	Sarcopenia	Mean	Mean difference	P-value
Kilocalorie (consumption)	Non-sarcopenia	1559.62 ± 446.68	523.99	0.001
	Sarcopenia	1035.63 ± 197.95		
Calorie deficit	Non-sarcopenia	438.46 ± 131.65	-105.08	0.005
	Sarcopenia	543.54 ± 120.39		

## Discussion

In this cross-sectional observational study, sarcopenia was observed in 24 (48%) patients with liver cirrhosis, with a predominance among men (men/women: 38 {76%}/12 {24%}). These findings align with previously reported prevalence rates in the literature. According to prior Indian studies based on CT assessments, sarcopenia prevalence in patients with cirrhosis was 36% and 56% [29,4]. Sarcopenia prevalence in cirrhosis ranged from 42% to 68% in other Asian countries [30]. Variations in racial characteristics, body size, and dietary habits may underlie differences in prevalence between Asian and Western populations [17].

A vicious cycle between sarcopenia and ectopic fat accumulation, mediated by mitochondrial dysfunction, proinflammatory cytokine release, oxidative stress, collagen deposition, extracellular matrix remodeling, and lifestyle factors, is known as lipotoxicity and provides a pathophysiological explanation for sarcopenia; increased disability, morbidity, and mortality can be caused by the exacerbation of lipotoxicity [31,32]. Lower BMI coupled with higher LFI is significantly associated with sarcopenia, as shown in the current study by comparison of BMI to LFI between patients with sarcopenia and non-sarcopenia.

Patients with sarcopenia tended to have higher CTP grades; however, the association was not statistically significant (χ² = 4.42; p = 0.109) in our study. Similarly, earlier studies have shown that sarcopenia is more prevalent in patients with cirrhosis with CTP class C functional status [33]. A uniform definition remains absent due to variability in measurement methods, but cross-sectional imaging via MRI or CT is considered the gold standard for sarcopenia assessment. Previous reports have shown that CT-derived PMI values of 295 mm²/m² in women and 356 mm²/m² in men can be defined as sarcopenia [17].

A strong association between PMI and sarcopenia is demonstrated, as the mean PMI was significantly lower in patients with sarcopenia than in individuals with non-sarcopenia (287.28 versus 502.4; p = 0.001). Sarcopenia can be reliably assessed with the total skeletal muscle index or the psoas muscle index, with a well-established correlation between total muscle area and psoas muscle area at L3 [34]. Psoas muscle area measurement alone is less cumbersome, does not require additional software, and provides an objective, reproducible method for diagnosing sarcopenia, which is observed in roughly half of patients with cirrhosis [17].

The ROC curve analysis for HGS as a predictor of sarcopenia revealed an AUROC of 1.000 and 0.903, indicating strong diagnostic accuracy in female (p = 0.004) and male (p = 0.001) patients; 17.3 kg and 26.1 kg were determined to be the optimal cut-off values of HGS for predicting sarcopenia, with sensitivities and specificities of 100% and 85.7% and 94.7% and 63.7% for female and male patients, respectively. The results are comparable to previous studies.

De et al. reported HGS as an independent predictor of sarcopenia with an AUROC of 0.73 in male patients with decompensated cirrhosis, using a cut-off of ≤31 kg [35]. Similarly, Salama et al. reported that an HGS cut-off of ≤28.6 kg differentiated patients with sarcopenia, with an area under the curve (AUC) of 0.879, sensitivity of 100%, and specificity of 66.7% [36].

HGS offers several advantages as a measure of muscle function and sarcopenia. It directly assesses muscle function; is inexpensive, widely available at the bedside, and radiation-free; and requires minimal training, unlike CT-based tools. Ascites or fluid retention does not affect HGS, unlike dual-energy X-ray absorptiometry or bioelectrical impedance analyses [3].

HGS has been shown to reflect body cell mass depletion and correlates with mortality in patients with cirrhosis and transplant waitlist patients [35]. HGS may better reflect patient mobility, physical activity, and overall functional status by assessing muscle function, as demonstrated in geriatric populations [37]. It is also a superior predictor of clinical outcomes compared to muscle mass alone [38], and its correlation with muscle mass is well-established in older adults [39].

Morbidity and mortality in patients with cirrhosis are adversely impacted by sarcopenia and frailty [40]. The ROC analysis for the LFI in this study showed an AUROC of 0.985, indicating excellent diagnostic performance (p = 0.001). An LFI cut-off of 4.2 predicted sarcopenia with 91.7% sensitivity and 92.3% specificity. Kaps et al. similarly reported that LFI, particularly the chair stand subtest, effectively predicted rehospitalization in patients with cirrhosis, with an LFI cut-off of >4.62 discriminating high-risk individuals [41].

National Family Health Survey 2015-2016 data indicates underweight prevalence of 19.6% among Indian men and 22.4% among Indian women [42]. These figures highlight a substantial burden of malnutrition, which may partly explain the high prevalence of sarcopenia in cirrhosis [11]. In this study, patients with sarcopenia had lower calorie intake and experienced a greater calorie deficit. Anand and Saraya emphasized that sarcopenia and malnutrition are not synonymous; malnutrition assessment requires the evaluation of dietary deficiencies, anthropometry, body composition, and functional status. Importantly, even in the absence of sarcopenia, patients with cirrhosis should undergo malnutrition screening [11].

Limitations

This study has a few limitations. First, the data was collected from a single tertiary care center. Second, the sample size is moderate, so the results should be considered a proof of concept. The proportion of the female population was low in our study. Larger, multicenter studies are needed to establish robust sarcopenia cut-offs for patients with cirrhosis. Finally, assessing L3 skeletal muscle mass index and its correlation with psoas muscle parameters would further clarify the role of psoas muscle measurement.

## Conclusions

In conclusion, the prevalence of sarcopenia was 48% in patients with liver cirrhosis in our study setting. The optimal cut-off values of HGS and LFI for predicting sarcopenia were determined to be 17.3 kg (women) and 26.1 kg (men) and 4.2, respectively, with good sensitivity and specificity. Indian-specific cut-offs should be used to define sarcopenia, as Western cut-offs may lead to overestimation. Nevertheless, these findings should be considered in the context of the study design, and more robust cut-offs need to be established through larger, multicenter studies in the future.
